# Anisotropic fluoride nanocrystals modulated by facet-specific passivation and their disordered surfaces

**DOI:** 10.1093/nsr/nwaa042

**Published:** 2020-03-13

**Authors:** Ziyu Yang, Huihui Zhang, Junjie Xu, Renzhi Ma, Takayoshi Sasaki, Yu-Jia Zeng, Shuangchen Ruan, Yanglong Hou

**Affiliations:** 1 Beijing Key Laboratory for Magnetoelectric Materials and Device (BKLMMD), Beijing Innovation Center for Engineering Science and Advanced Technology (BIC-ESAT), Department of Materials Science and Engineering, College of Engineering, Peking University, Beijing 100871, China; 2 College of Physics and Optoelectronic Engineering, Shenzhen University, Shenzhen 518060, China; 3 International Center for Materials Nanoarchitectonics (WPI-MANA), National Institute for Materials Science (NIMS), Tsukuba 305-0044, Japan

**Keywords:** solution synthesis, fluorides nanostructures, magnetic properties, exchange anisotropy, surface effect

## Abstract

Rutile-type fluorides have been proven to be active components in the context of emerging antiferr-omagnetic devices. However, controlled synthesis of low-dimensional, in particular two-dimensional (2D), fluorides in a predictable and deterministic manner remains unrealized because of a lack of efficient anisotropic control, which impedes their further development in reduced dimensions. We report here that altered passivation of {110} growing facets can direct the synthesis of rutile-type fluoride nanocrystals into well-defined zero-dimensional (0D) particulates, one-dimensional (1D) rods and 2D sheets in a colloidal approach. The obtained nanocrystals show positive exchange bias and enhanced magnetic transition temperature from the coexistence of long-range antiferromagnetic order and disordered surface spins, making them strong alternatives for flexible magnetic devices and sensors.

## Teaser

The altered passivation of growing facets can direct the colloidal synthesis of rutile-type fluorides nanocrystals into 0D particulates, 1D rods, and 2D sheets.

## INTRODUCTION

**Figure 1.**
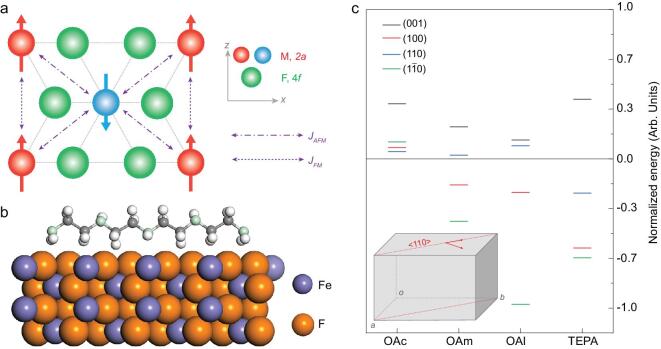
(a) Magnetic interactions in rutile-type MF_2_ (M = Mn, Fe, and Co) in a [010] view direction. (b) Side view of the geometry-optimized configuration of TEPA molecule at FeF_2_ (001) surface. Color code: dark grey, C; cyan, N; white, H. (c) Calculated adsorption energies of OAc, OAm, OAl and TEPA molecules at FeF_2_ (001)-grey, (100)-red, (110)-blue and $(1\bar{1}0)$-green surfaces. Inset shows the <110> growth direction in a sketched lattice.

When magnetic materials are nanometric at least in one dimension, the surface effect often dominates the static and transport behaviors as a result of broken translation symmetry [1]. The unperfect surface spin coordination and perturbations in spin-spin correlation length make low-dimensional magnetic materials an ideal platform for exploring the copious magnetism in reduced dimensions, especially those that are two-dimensional (2D) [2–4]. Solution processing followed by patterning or assembling these materials to integrated objects poses a conceptual flatland for mechanically flexible, engineerable and biocompatible devices with complex functionalities [5,6]. Recent fast progress in spintronics has brought antiferromagnetic (AFM) counterparts to an emerging field, besides the traditional notion of magnetization reversal by quantum tunneling [7,8]. As a typical long-range AFM order, the rutile-type fluorides MF_2_ (M = Mn, Fe, and Co) have proven very useful in the context of antiferromagnetic spintronics, especially in the THz range with optical manipulation [9]. Described in terms of the Stoner-Wohlfarth model, two magnetic sublattices orient collinearly with uniaxial magnetic anisotropy in these materials. The antiparallel aligned moments along the tetragonal *c* axis are defined in Fig. 1a, where antiferromagnetic *J_AFM_* and ferromagnetic *J_FM_* exchange interactions alternate between nearby moments with a negative Weiss molecular field [10].

However, unlike the van der Waals materials with weakly stacked planes, the micromechanical and more common methodology ‘liquid exfoliation’ is not easily accessible for rutile-type fluorides [11,12]. Some instructive understandings remain unclear because of fundamental hindrance of obtaining these materials in low-dimensions, such as how size matters come to play, and how the subtle interplay between the surface spin arrangement and phase transition operates. It is challenging to initiate and sustain the solution processability of fluorides in a predictable, controlled and deterministic manner, and to clarify their role as a complex mesoscopic system. Considering the tetragonal geometry structures of rutile fluorides, a preferential 1D structure is plausible, whereas the 0D and 2D structures are somewhat more difficult to establish. This is further supported by the fact that most reported fluorides nanocrystals in solutions are 1D oriented, while the 0D and 2D products are scarce [13,14]. Although an inherently anisotropic crystalline structure serves as the driving force for asymmetrical growth, the sifted organic ligand molecules will play a crucial role in determining the oriented growth by capping specific surfaces [15–17]. In this work, we show that asymmetric passivation by elected surfactants can drive the normally 1D fluoride nanocrystal growth into 0D or 2D structures from the perspective of facet-specified adsorption. Moreover, the obtained nanocrystals exhibit anomalous hysteretic behavior and positive exchange anisotropy, in which the disordered surface spins generate robust enhancement of the transition temperature.

## RESULTS AND DISCUSSION

**Figure 2.**
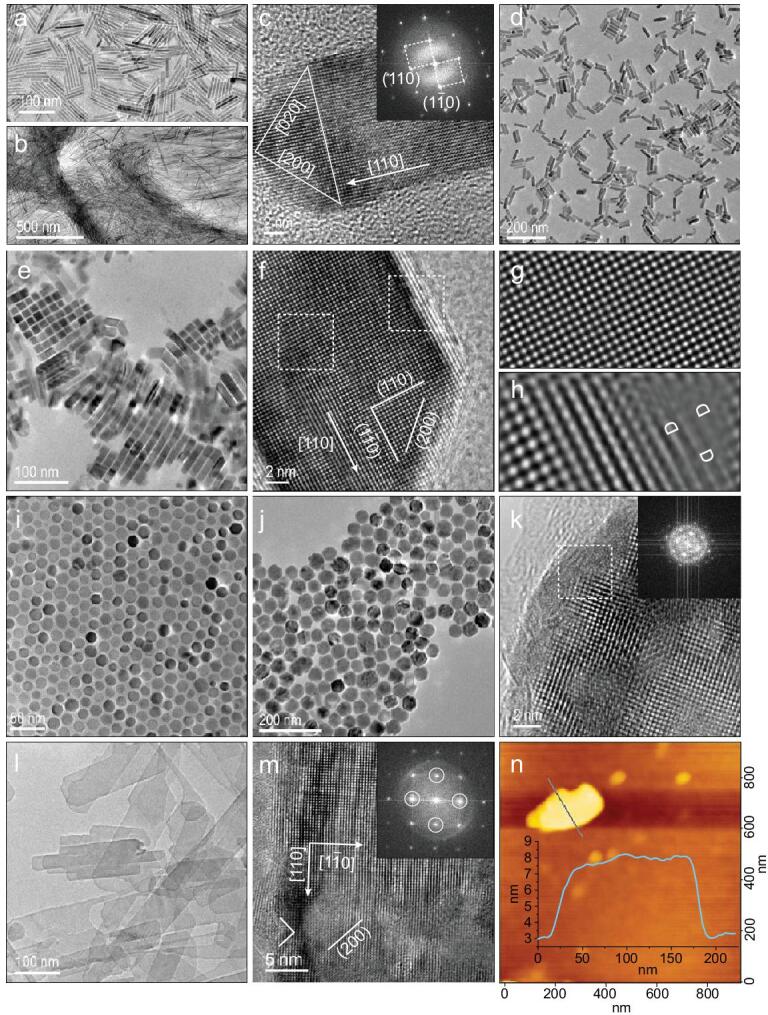
Morphological and structural analysis of varied FeF_2_ nanocrystals. (a, b) Bright-field TEM images of the typical 1D FeF_2_ rods with varying diameters and the high-resolution TEM image (c) showing the growth direction [110] determined by FFT. (d, e) 1D FeF_2_ rod with smaller AR value and assemblies on the water showing the transverse planes. (f–h) High-resolution TEM image of 1D FeF_2_ in (d) and inverse FFT images from the bulk (g) and corner (h) areas in (f), where ‘D’ represents distortions. (i–k) TEM and high-resolution TEM images of 0D FeF_2_ particulates using OAc. Inset is the corresponding FFT image of a local region labelled by white-framed area in (k). (l–n) TEM, high-resolution TEM and atomic force microscopy (AFM) images of the 2D FeF_2_ sheets synthesized with TEPA, with a thickness of ca. 5 nm. The FFT image shows that the growing directions are along <110>.

To clarify the complexity of altering the course of reactions, we use density functional theory (DFT) methods to consider the role of surfactants classified to acids (oleic acid, OAc), amines (oleylamine, OAm, tetraethylenepentamine, TEPA) and alcohols (oleyl alcohol, OAl), as shown in Fig. 1b,c and Supplementary Fig. S1. According to our calculations, preferential capping on the (001) facet is found in all the evaluated molecules, revealing that the growth direction of the *c*-axis is impeded. Besides, the asymmetric adsorption of {110} facets with subsequent blocking serves as the origin of rod formation in a direction perpendicular to the (110) or $(1\bar{1}0)$ facet when OAc, OAm and OAl molecules are used. The experimental results are in good agreement with theoretical predictions, where FeF_2_ nanocrystals with well-defined crystalline orientations are obtained (see Fig. 2 and Supplementary Fig. S2). Powder X-ray diffraction patterns confirm a rutile-type structure with space group *P*4_2_/*mnm*, 136 (ICSD code: 073730), as shown in Supplementary Fig. S3.

The 1D FeF_2_ rods with a varied aspect ratio (AR) are depicted in Fig. 2a–h, in which a single-crystalline structure with crossed {110} lattice planes displaying an angle of 45° relative to the rod edges is identified (Fig. 2c and f). This is in accordance with the theoretical descriptions that the rods grow parallel to the <110> orientation with exposing {001} facets. A typical water-gas interface-based self-assembly pattern on a copper grid is shown in Fig. 2e, in which the rectangular cross-sections further confirm the predicated <110> orientation. Inverse FFT images suggest that the inner bulk lattices are highly ordered, as evidenced by the local area labelled with a dotted box in the high-resolution TEM image (Fig. 2f–h), while highly strained arrangement of atoms is observed near the surface (denoted as ‘D’, extracted from the white dotted box in Fig. 2f). During the crystal growth process, the nanocrystals tend to grow fast perpendicular to the most energetically unfavorable facets, thus {200} facets are highly active and are preferentially consumed during the growth. Besides, the concentration of OAm/OAc and temperature of the reactions modulate the growth process effectively, thus regulating the aspect ratios. The OAc molecule shows effective adsorption to all the evaluated facets, indicating the possible formation of nearly spherical particulates if growth kinetics at various stages of the reactions is controlled, such as ceasing the reaction within a few minutes. The spherical particulates with varying diameters are depicted in Fig. 2i–k and Supplementary Fig. S2, showing a rutile-type monocrystalline structure corroborated by the selected-area electron diffraction pattern. As predicted in the DFT calculations, the TEPA molecule plays a key role in formation of 2D FeF_2_ sheets, from the fact that weak adsorption ability is found for (100), (110) and $(1\bar{1}0)$ facets but strong affinity is found for the (001) facet. No restraint is expected in the <110> direction, whereas the growth should be robustly confined perpendicular to the (001) facet. The 2D sheets have a lateral dimension of several hundred nanometers and a thickness of ca. 5 nm, as shown in Fig. 2l–n. High-resolution TEM imaging confirms the <110> growth direction with pronounced (200) facets, and small terraces of {200} planes are also present with (001) exposing planes identified by the 90° angle at the crystalline edge (Fig. 2m). The growth of CoF_2_ and MnF_2_ nanocrystals shows analogous behavior during the reaction courses, reinforcing the idea postulated in the preceding section, see Supplementary Figs S4–S9.

**Figure 3.**
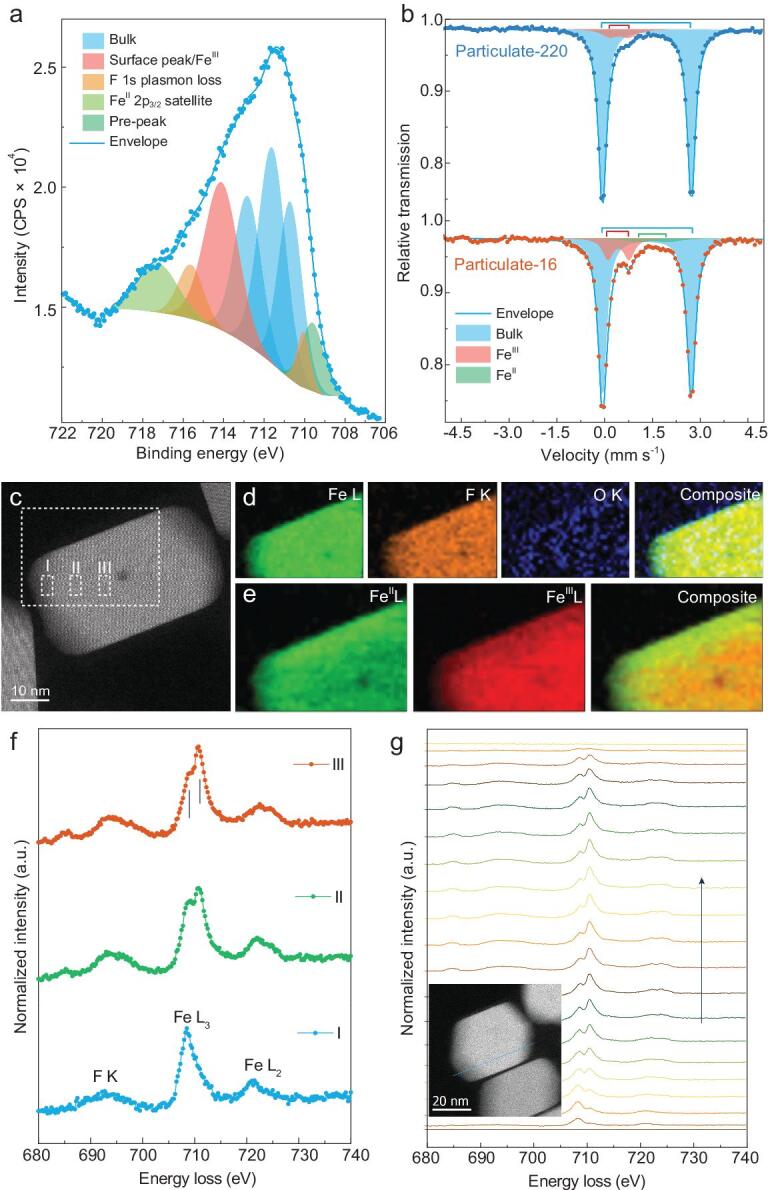
(a) Shirley-type backgrounded Gupta-Sen multiplet fittings for Fe 2*p_3/2_* of 1D FeF_2_ rods. (b) Q.S. doublet fittings for the Mössbauer spectra. (c) HAADF-STEM image and the corresponding elemental maps (d) of Fe L, F K and O K-edge from the selected area of (c) for 1D FeF_2_ rods. (e) STEM-EELS elemental mapping of Fe^II^ and Fe^III^ for 1D FeF_2_ rods. (f) EELS spectra of F K, Fe L_3_ and L_2_-edges collected from the selected spots of (c). (g) Spectrum evolution of Fe L_3_-edge decomposed to Fe^II^ and Fe^III^. The blue line shown in the inset indicates the scanning path where EELS data are collected.

A high-resolution X-ray photoelectron spectrum of Fe 2*p*_3/2_ is analyzed using calculated Gupta-Sen multiplets to discriminate the surface state [18,19]. A single satellite peak including *t_2g_* and *e_g_* 3*d* transitions, and a single low-intensity peak on the low-binding energy (BE) side are introduced to determine the Fe^II^ species [20]. The results follow Gupta-Sen predictions well, allowing for tiny deviations (Fig. 3a). Apart from the F 1 *s* plasmon loss peaks, a large peak containing high-BE surface structure and high-spin Fe^III^ species is deduced, which is possibly derived from the decreased coordination and hydration of the surface. Energy separation between the Fe 2*p*_3/2_ main peak center of gravity (CG) and the shake-up satellite *ΔE*_(satellite-2_*_p_*_peak CG)_ = 5.9 eV is much lower than the reported value of Fe^II^-F (6.5 eV) and Fe^III^-F (8.7 eV) bonds, indicating decreased electronegativity of the ligand because of possible Fe-O bonding derived from the atmospheric oxygen [21]. Furthermore, the lowered F 1 *s* and Fe 2*p*_3/2_ peak CG reveals increased shielding of the Fe nucleus (Supplementary Fig. S10) [22,23]. Electrostatic perturbation arising from a charge difference is much higher than the elastic perturbation of the lattice arising from ion size effect, thus oxygen is readily detected in ionic fluorides possibly from an energetically favored hydrolysis process. Hence, *T_2_* type trivalent substitutional M^III^···O^II^ complexes will form to provide sufficient compensating lattice energy to stabilize the O^II^ substitutional [24,25].

Recoil-free ^57^Fe Mössbauer spectrometry provides further evidence of possible oxygen trapping and the existence of the *T_2_* complex, as depicted in Fig. 3b and Supplementary Fig. S10c. In all cases, a sum of Lorentz doublets is applied to refine the experimental points with their related contributions assigned unambiguously to the bulk and surface component: the isomer shift (I.S.) is exactly the same for the bulk entities, while the interfacial Fe^II^ and Fe^III^ proportions are rather dependent on the samples under an octahedral symmetry [26]. A typical spectrum of 16 nm particulate is fitted with three quadrupole splitting (Q.S.) doublets with broadened spectral linewidth, indicating the existence of a large concentration of grain boundaries with a different local arrangement of atoms [27]. The characteristic behaviors of (i) decreased value of I.S. of the Fe^III^ doublets with reduced diameters and (ii) higher line-width of Fe^II^ and Fe^III^ doublets than that of the bulk suggest that the Fe-F bonds are stretched and that Fe-O-F mix-bonded Fe^II^/Fe^III^ doublets originate from the atoms in the grain boundaries with structural relaxations. A decreased Q.S. value of Fe^II^ is found to accompany the reduction of Fe^II^/Fe^III^ ratio as the size of the nanocrystals decreases, further reinforcing the idea that Fe^II^ and Fe^III^ are in terms of the non-stoichiometric Fe^II^-F and Fe^III^-F octahedra structures, as shown in Supplementary Table 1 [26]. High angle annular dark-field scanning transmission electron microscopy images (HAADF-STEM) and their corresponding elemental maps clearly show that oxygen-enriched phases mainly distribute along the deficient surface, as depicted in Fig. 3c,d. Broad dichotomic Fe L_3_-edge spectra of electron energy loss spectroscopy (EELS) is obtained, revealing a variation of Fe^II^/Fe^III^ in spatial distribution (Fig. 3f). The Fe^II^ suffuses across the whole particle while Fe^III^ is incorporated into the rutile-type lattices, as evidenced by the evolutionary change of Fe L_3_-edge intensity and corresponding elemental maps (Fig. 3e–g). Line EELS profiles promise qualified estimations of distortions in the interior and at the surface, which is in gratifying agreement to the theory proposed by Lidiard, that is substitutional entities diffuse into ionic crystals by forming complexes with vacancies on the same sublattice [28].

**Figure 4.**
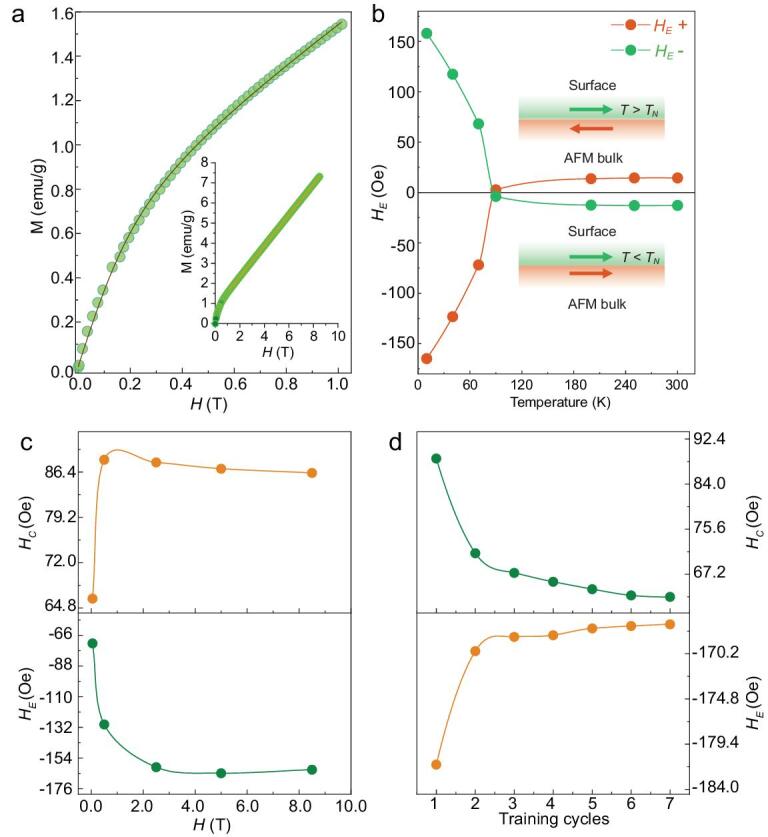
Magnetization characterizations of 0D FeF_2_ particulate with an average diameter of 24.0 ± 1.0 nm. (a) Typical initial magnetic isotherm of 300 K obtained from dc magnetization. The solid line is a fit of the modified Langevin function. Inset shows the extrapolation to *H* = 0 under high applied field up to 8.5 T. (b) Field cooling under ± 5 T field: thermal behavior of the exchange anisotropy field *H_E_*. (c) *H_E_* and coercive field *H_C_* as functions of the cooling field *H_FC_*. (d) Detail of the magnetic training effect in *H_C_* and *H_E_*. Lines are guides to the eye.

Low field dc susceptibility curves *χ* = *M/H* (measured in an applied field of 50 mT) of the 0D FeF_2_ particulate with an average diameter of 24.0 ± 1.0 nm during heating after zero field cooled-field cooled conditions exhibit strong Néel transitions, with a critical temperature *T_N_* of ca. 89.9 K, which is higher than that of the bulk (78 K, see Supplementary Fig. S11a). Inverse susceptibility in the high-temperature regime follows Curie-Weiss law *χ* = *C*/(*T* – *Θ_P_*) with *Θ_P_*<0, indicating the presence of robust long-range AFM order in the system. Isothermal magnetization curves are collected in fields of up to 8.5 T, as depicted in Fig. 4a. The initial magnetic isotherm is fitted with a modified Langevin expression including a linear contribution as

(1) \begin{eqnarray*}M\!\left( H \right) &=& {M_{s\ }} \cdot \left[ {{\textit coth}\!\left( {A \cdot \ H} \right)\ - \frac{1}{{\left( {A \cdot H} \right)}}} \right]\nonumber\\ &&+\, \chi H,\end{eqnarray*}

where *M_s_* is the saturation magnetization and *A* = *μ_C_*/*k_B_T*, *μ_C_* represents the uncompensated spins [29]. An intrinsic magnetization of 0.77 emu/g is deduced via extrapolating *M-H* curves to *H* = 0, indicating a magnetic moment per particle *μ_P_* = 4942 *μ_B_*, which is in agreement with the Langevin fitted parameter *μ_C_* = 4823 *μ_B_*.

In a monodomain AFM cluster containing *N* ions, each of moment ***m***, the moment pointing in random directions is $\surd {\rm{N}}$***m***, thus a rough value of *μ_C_* = 3080 *μ_B_* (*S* = 4/2) is estimated via Néel's model [30,31]. The predicted value is much lower than that obtained by the experimental measurements even if considering the Fe^III^ spins (*S* = 5/2), suggesting another moment ***m*** aside from the random arrangement of uncompensated sites in the AFM system. A thermal dependent exchange bias *H_E_* (the magnitude of the field offset from the origin in the exchange anisotropy terminology) is identified, as depicted in Fig. 4b and Supplementary Figs S11, S12. A decreased *H_E_* with increased temperature below *T_N_* is observed, indicating thermal diminished interfacial exchange coupling. However, the anomalous positive *H_E_* when *T* > *T_N_* with a magnitude 8% of that at 10 K proves the existence of pinned FM spin structure induced by thermal evolution [32]. A *ferromagnetic* FM-AFM interaction with the AFM surfaces coupling to the external cooling field *H_FC_* results in the usually negative *H_E_*. Considering the magnetically uncompensated spins of the AFM system, the cooling field breaks the two-sublattice symmetry. Thus, an *antiferromagnetic* FM-AFM interaction frustrated by the *ferromagnetic* coupling between the AFM surface spins and the cooling field can induce positive exchange anisotropy *H_E_* under a temperature range *T* > *T_N_* [32]. The ${|}$*H_E_*${|}$ monotonically increases from *H_FC_* = 500 Oe to the saturated value at *H_FC_*$ \approx $2.0 T, with a magnitude of 132% larger than that at low cooling field *H_FC_*. The *H_C_* changes with analogous behavior, as depicted in Fig. 4c. A decrease of 7% of *H_E_* is identified by training of seven consecutive hysteresis loops (Fig. 4d). Note that the positive *H_E_* is almost constant under the temperature range *T*${\rm{ > \ }}$*T_N_*, suggesting that the pinning effect exerts around *T_N_* in a short correlation length, which serves as the dominate pillar accounting for the anomalous exchange anisotropy [33].

**Figure 5.**
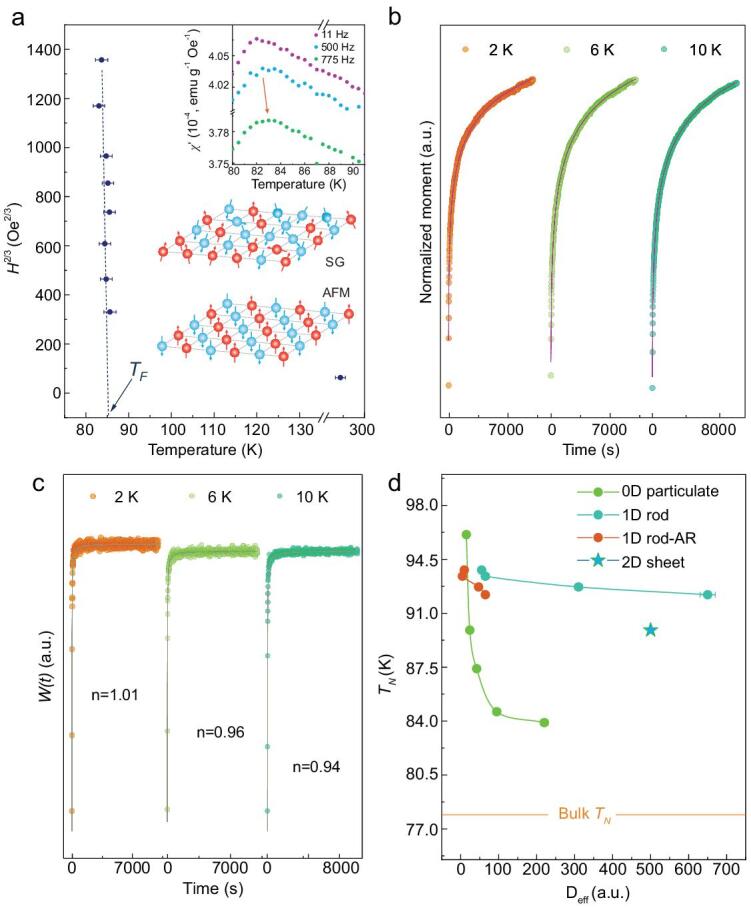
Dynamics of 0D FeF_2_ particulate with an average diameter of 24.0 ± 1.0 nm. (a) Field dependence of the spin-glass transition temperature *T_F_* indicating the *A-T* line, i.e. *ΔT_F_* ∝ *H*^2/3^. Inset shows the real part of the ac susceptibility *χ’(T)* recorded at different frequencies *ν* and at an ac field *H_ac_* = 8 Oe, dc field *H_dc_* = 500 Oe. (b) Relaxation of the ZFC magnetization at varied temperatures of 2, 6 and 10 K, and their corresponding rate curves (c). (d) Dimensional dependence of the transition temperature *T_N_* for varied FeF_2_ nanocrystals with evaluated diameters D_eff_. The marked lines are guides to the eye.

The observation of field-dependent irreversible magnetization further confirms the conclusions, where *T_F_* is defined as the bifurcation of ZFC and FC magnetization, that is Δ*M* = *M_FC_* – *M_ZFC_* becomes different from zero, as depicted in Fig. 5a and Supplementary Fig. S13. The power dependence, $\Delta {T_F}\ {H^{2/3}}$, corresponding to the de Almeida-Thouless (*A*-*T*) line is given by

(2) \begin{equation*}{H_{AT}}/\Delta J \propto {\left( {1 - T/{T_F}} \right)^{3/2}},\end{equation*}

a characteristic temperature of ca. 85 K is identified by the *H^2/3^* law extrapolation, indicating the onset of the freezing process [34,35]. The ac susceptibility *χ’* measurement exhibits a pronounced anomaly at ca. 83 K, in accordance with the *T_F_* derived from dc magnetization. The relative peak shift per decade of frequency, *δT_F_* = *ΔT_F__/_*[*T_F_*·*Δ*(log_10_*ν*)], where *ΔT_F_* = *T_F_* _(_*ν_1_*_)_–*T_F_*_(_*ν_2_*_)_, *Δ*(log_10_*ν*) = log_10_*ν_1_−* log_10_*ν_2_*, also known as the Mydosh parameter, is identified to be 0.0065, which is in the range of observed values in the spin-glass categories [36]. It is worth mentioning that the strong irreversibility remains up to the highest field used (5 T), confirming that the disorder is confined in a well-defined surface layer [35]. Note that *T_N_* of ca. 89.9 K is slightly higher than the freezing temperature *T_F_*. Thus, a preferred orientation is imposed upon the surface spin-glass-like spins during the FC process. Upon heating up to the Néel transition, the AFM core experiences the excess field from the frozen surface layer, generating a pinned FM spin structure *antiferromagnetically* coupled to the AFM core that results in the observed positive exchange anisotropy. Extra energy is required to switch the spins pinned by the freezing layer, resulting in a strong increase of *H_C_* around the *T_F_*, as depicted in Supplementary Fig. S14a.

The relaxation process further reinforces the surface disordered model, where the time revolution of magnetization is recorded after a lapse of waiting time *t* = 3600 s (Fig. 5b). A standard stretched exponential function in the form of

(3) \begin{equation*}M(t) = {M_0} - {M_g} \cdot {\textit exp}\left[ { - {{\left( {\frac{t}{\tau }} \right)}^\beta }} \right],\end{equation*}

is exerted, where *M_0_* represents the intrinsic magnetization of the surface layer, and *M_g_* relates to the glassy component of magnetization [37]. The characteristic relaxation time *τ* is determined to be in the range of 4600 ∼ 5300 s, while the stretching exponent *β* is identified to be 0.10 ∼ 0.23, falling in the range of obtained values for systems evolving through a distribution of energy barriers [36]. The relaxation rate *W(t)* theoretically determined using Monte Carlo simulations by Ulrich *et al.* is further examined, relating to the magnetization by the form of

(4) \begin{equation*} M \,(t) = {e^{ - \mathop {\small {int}} \limits_0^t W (t)dt}}, \end{equation*}

where *W(t)* is in a power-law decay above some crossover time *t_0_*, *W*(*t*) = *At^−^^n^*, *t* ≥ *t_0_*, the pre-exponential factor *A* is constant, while *n* is an exponent function of temperature in the relaxation process [37,38]. The exponent *n* is identified by fitting the relaxation rate *W(t)* and the corresponding logarithmic presentation log*W(t) vs* log*t*, to be 1.01, 0.96, 0.94 for 2, 6 and 10 K, respectively, which confirms the cluster spin-glass behavior in the system, as depicted in Fig. 5c and Supplementary Fig. S14.

Surface effects are sensitive to any non-stoichiometry and are more pronounced with decreasing size, which is also the reason for the absence of cusp in ac susceptibility data [39–41]. The translational symmetry breaking of the AFM lattice and unsaturated bonds generating random fields or interactions may be responsible for the surface spin freezing behavior. Distortions on the atomic scale and heterogeneity also account for the spin frustration. Generally, the surface distortion in nanostructured materials results in a decreased transition temperature because of the reduced exchange constant and limited correlation length [42]. Nevertheless, we find an enhanced magnetic transition temperature for all the obtained samples despite their topological differences, as depicted in Fig. 5d. In our case, the core spins are in an AFM network while surface spins freeze like a spin-glass, generating a pinned FM moment *M_F_* that has profound effects on the transition of the entire nanocrystal. Considering the order parameters in the framework of Landau theory, where structure parameter *P_S_*, AFM order parameter *P_A_* and *M_F_* are included in the first approximation, the free energy *G_L_* is given by an expansion:

(5) \begin{eqnarray*} &&{G_L}\ &=& \ {G_{L0}}\ \ + \ \frac{1}{2}\Gamma P_A^2\ + \ \frac{1}{4}BP_A^4\ + \ {P_S}{P_A}{M_F}\nonumber\\ &&+\, \ \frac{1}{2}AM_F^2,\end{eqnarray*}

where *B*$ > {\rm{\ }}$0, *A*$ > {\rm{\ }}$0, *Γ =* (*T* – *T_bulk_*)*/T_bulk_* is the reduced temperature [43,44]. Minimizing *G_L_* with respect to *P_A_* and *M_F_* by

(6) \begin{equation*}\frac{{\partial {G_L}}}{{\partial {P_A}}} = \ 0,\ \ \frac{{\partial {G_L}}}{{\partial {M_F}}} = \ 0,\end{equation*}

when in the critical point, *P_A_* = 0, hence the critical phase transition temperature is enhanced by *T* = *T_bulk_* · (1 +${{P}}_{{S}}^{{2}}$/*A*). Moreover, additional increasing of the diameters of the nanocrystals should decrease the transition temperature as the characteristic correlation length is approaching the value of the bulk, as identified in Fig. 5d.

## CONCLUSION

We have presented that altered passivation of growing facets can direct the colloidal synthesis of rutile-type fluoride nanocrystals into well-defined 0D particulates, 1D rods and 2D sheets in a predicted and controlled manner. The presence of TEPA molecules is essential in formation of 2D sheets by preferential packing of -NH_2_ ligands on the (001) facets and blocking them from further growth. A cluster spin-glass-like surface layer is identified from the disrupted translation symmetry at the surface, which exerts a pinned FM moment on the AFM core. Anomalous positive exchange bias *H_E_* and enhanced magnetic phase transition temperature are observed from the interactions between pinned FM moments and the associated structural order parameters, which are qualified within the framework of Landau theory. These high-quality fluoride nanocrystals are strong candidates for flexible antiferromagnetic devices and sensors. Moreover, we believe that this approach of anisotropic direction of the growing process will pave the way to solution synthesis of other low-dimensional halide nanocrystals for emerging spintronics, such as 2D FeCl_2_ and CrI_3_.

## MATERIALS AND METHODS

The controlled synthesis of rutile-type fluorides MF_2_ (M = Mn, Fe, and Co) nanocrystals is realized both through ‘*one-pot’* and *‘conversion-chemistry’* based methods, where metallic or oxide nanocrystals are used as conversion seeds. NH_4_F, NH_4_HF_2_ and ammonium trifluoroacetate (ATF) were utilized as the fluorine source, while their ability to provide F^-^ is in a sequence: NH_4_HF_2_>NH_4_F>ATF. *Note that the fluorine-contained chemicals are often of acute toxicity, and may cause severe skin burns and eye damage.* The comprehensive details, chemicals, characterizations and theoretical methods are in the Supplementary data.

## Supplementary Material

nwaa042_Supplemental_File
