# Supplementary material for: Anisotropic fluoride nanocrystals modulated by facet-specific passivation and their disordered surfaces
Source: Natl Sci Rev. 2020 Mar 13;7(5):841–8. doi: 10.1093/nsr/nwaa042 (PMC8288850; doi:10.1093/nsr/nwaa042)
Supplement: nwaa042_Supplemental_File [file nwaa042_supplemental_file.docx]

Supplementary Data

**Anisotropic Fluoride Nanocrystals Modulated by Facet-specific Passivation and Their Disordered Surfaces**

Ziyu Yang^1^, Huihui Zhang^2^, Junjie Xu^1^, Renzhi Ma^3^, Takayoshi Sasaki^3^, Yu-Jia Zeng^2^, Shuangchen Ruan^2^, Yanglong Hou^1,*^

^1^Beijing Key Laboratory for Magnetoelectric Materials and Device (BKLMMD), Beijing Innovation Center for Engineering Science and Advanced Technology (BIC-ESAT), Department of Materials Science and Engineering, College of Engineering, Peking University, 100871, Beijing, China.

^2^College of Physics and Optoelectronic Engineering, Shenzhen University, 518060, Shenzhen, China.

^3^International Center for Materials Nanoarchitectonics (WPI-MANA), National Institute for Materials Science (NIMS), Namiki 1-1, Tsukuba, 305-0044, Ibaraki, Japan.

∗Corresponding authors.

E-mails: [hou@pku.edu.cn](mailto:hou@pku.edu.cn)

*The supporting information contains the following sections:*

**Methods**

*1. Chemicals.*

*2. Characterizations.*

*3. Synthetic procedures.*

*4. Theoretical methods.*

**Supplementary Figures**

*Figure 1-14*

**References**

**Methods**

1. ***Chemicals***

All the chemicals were used without additional purification and were performed under Ar/N_2_/vacuum lines utilizing a homemade heating apparatus, four-neck bottles (Synthware) and a glove box (MIKROUNA). Ammonium Fluoride (NH_4_F, 98%), 1-Octadecane (ODE, 90%), Oleic Acid (OAc, 90%), Oleyl Alcohol (OAl, 80-85%) were purchased from Alfa Aesar; Diphenyl Ether (99%), Iron Pentacarbonyl (>99.99%), Cobalt Carbonyl (90%), Cobalt (II) Acetylacetonate (99%), Tetraethylene Glycol (TEG, 99%), Tetraethylenepentamine (TEPA, technical grade), Benzyl Ether (98%), and Ammonium Trifluoroacetate (98%) were purchased from Sigma-Aldrich; Oleylamine (OAm, 98%), Manganese (II) Acetylacetonate (99%), NH_4_Br (99%), Ammonium Hydrogen Difluoride (NH_4_HF_2_, ≥98.5%) were purchased from J&K Chemicals; Iron (III) Acetylacetonate (99%) was purchased from Acros; Ferrous Acetylacetonate (99%) was purchased from Adamas-beta^®^.

1. ***Characterizations***

The FEI Tecnai T20 microscope (200 kV) and F30 microscope (300 kV) were performed to obtain the traditional bright-field images, energy-dispersive X-ray spectra (EDS) and selected area electron diffraction (SAED) patterns. Electron energy loss spectroscopy (EELS) spectra and elemental mapping images were collected on Titan^™^ Themis scanning transmission electron microscope (STEM). A PANalytical X’Pert^3^ Powder X-ray diffractometer equipped with Cu-K*α* radiation at 40 kV and 40 mA was used to obtain the Powder X-ray diffraction (PXRD) patterns. An Imaging Photoelectron Spectrometer (Axis Ultra DLD, Kratos Analytical Ltd.) using monochromatized Al K*α* anode (Al K*a*, *hv* = 1486.7 eV) along with the pressures in the analysis chamber of 10^-8^~10^-9^ torr was utilized to perform the X-ray photoelectron spectroscopy (XPS) measurements. All the collected spectra were calibrated with contaminated C 1*s* peak at 284.8 eV, and were analyzed using CasaXPS software (2.3.12 Dev7). Static and dynamical magnetic susceptibility data were collected with the Physical Property Measurement System (PPMS^®^DynaCool^™^, Quantum Design) and the Magnetic Property Measurement System (MPMS^®^3, Quantum Design) with field up to 8.5 T. Atomic force microscopy (AFM) images were obtained via a NanoNavi Nanocute Microscopy with a self-sensitive micro cantilever PRC-DF40P (468.4 kHz, SII NanoTechnology Inc.).

1. ***Synthetic Procedures***

The controlled synthesis of fluorides nanocrystals is realized both through ‘*one-pot*’ and ‘*conversion-chemistry*’ based methods, where metallic or oxide nanocrystals are used as conversion seeds. NH_4_F, NH_4_HF_2_ and ammonium trifluoroacetate (ATF) were utilized as the fluorine source, while their ability to provide F^-^ is in a sequence: NH_4_HF_2_>NH_4_F>ATF. *Note that these fluorine contained chemicals are often of acute toxicity, and cause severe skin burns and eye damage.*

***Metallic Fe and Co Nanocrystals:*** Iron particulates were prepared according to our previous report with minor modifications.^[1]^ Briefly, 20 ml ODE, 0.1 mmol NH_4_Br and 0.3 ml OAm were mixed and degassed under a gentle H_2_+Ar (5.5% v/v) flow for 1 hr in a four-neck flask. The solution was then heated to 100°C and kept at this temperature for 2 hrs before it was further heated to 180°C to fully remove the organic impurities. After that, 5 mmol Fe(CO)_5_ was injected to the reaction mixture and kept there for 30 mins. A colour change from salmon to brown then to the black of the solvents were observed after ca. 1 min, indicating the decomposition of Fe(CO)_5_ and the formation of *bcc*-Fe NPs. If the procedure was conducted without NH_4_Br, amorphous Fe particulates would be obtained. The synthesis of Co particulate was almost the same except that Co_2_(CO)_8_ was used as precursors, and the thermal decomposing temperature was elevated to 207°C. Both the Fe and Co nanocrystals were washed by hexane and ethanol (1:1, v/v, 27 ml) and were centrifuged at 11 000 g for 11 mins.

***Fe_3_O_4_/CoO/MnO Nanocrystals:*** A thermal decomposing of M(II)(acac)_2_ (M=Fe, Co, Mn) salt process was used to prepare the oxides seeds. Typically, 1 mmol M(II)(acac)_2_ (M=Fe, Co, Mn) salt was added to a mixed solution of 7 ml OAm and 7 ml benzyl ether. The mixed solution was heated to 90°C and kept there for 2 hrs under a H_2_-Ar (5.5% v/v) flow untill the solution turned transparent. After that, the solution was heated up to 120~250°C (typically 120, 200, 250℃) under a H_2_-Ar (5.5% v/v) atmosphere and kept there for 30 mins ~ 6 hrs (typically 0.5, 1.6, 2.4, 3.8, 6 hrs). The products were washed with hexane and ethanol (1:2, % v/v) for three times, and centrifuged at 11 000 g for 11 mins.

***Conversion-chemistry Based Method:*** Typically, an equivalent 1 mmol of the seeds dispersed in 5 ml hexane was injected to a solution containing 13.5 ml ODE, 2.5 mmol F^-^ source. Different surfactants of OAc, OAm, OAl, TEPA and TEG with varying concentrations were added respectively. The related concentrations of the surfactants were in the range of 0.3% v/v~100% v/v (typically 0.3%, 4%, 6%, 9%, 15%, 24%, 38%, 62%, 100%). Before heated up to the set temperature, the mixed solutions were magnetically stirred under a H_2_+Ar (5.5% v/v) line at 90°C for 2 hrs. Then the system was further heated up to 160 °C~340°C (typically 160, 270, 340°C) and kept there for 30 mins~2 hrs (typically 0.5, 1.4, 2 hrs). After cooling to room temperature, the products were washed with hexane and ethanol for three times and collected by centrifugation of 11 000 g for 11 mins. The coloured precipitates (FeF_2_-salmon, CoF_2_-purple, MnF_2_-milk) were dispersed in methylbenzene with several droplets of OAm for further characterization, except that the TEPA/TEG capped products were dispersed in ethanol.

***One-pot Synthesis of MF_2_ Nanocrystals:*** A thermal decomposing of M(II)(acac)_2_ (M=Fe, Co, Mn) salt process under the existence of an excess amount of F^-^ source (F:M=2.5:1, mole ratio) was utilized to control the size and dimensionality of MF_2_ nanocrystals. Typically, 13.5 ml ODE, 1 mmol M(II)(acac)_2_ (M=Fe, Co, Mn) salt, and 2.5 mmol NH_4_F were mixed and degassed under a gentle H_2_-Ar (5.5% v/v) flow for 1 hr in a four-neck flask. Varied surfactants with concentrations the same as used in the ‘*conversion-chemistry*’ section were added (0.3% v/v~100% v/v, typically 0.3%, 4%, 6%, 9%, 15%, 24%, 38%, 62%, 100%). The solution was magnetically stirred at 90°C for 2 hrs to fully remove the organic impurities. After the mixed solution turned transparent, the system was further heated to the set temperature (160°C~340°C, typically 160, 270, 340°C) and kept there for 30 mins~2 hrs (typically 0.5, 1.4, 2 hrs). Hexane and ethanol (30 ml, 1:2, v/v) were added to precipitate the product after the system was cooled to room temperature via centrifugation (11 000 g for 11 mins). After the washing procedure was repeated for three times, the final product was dispersed in methylbenzene with several droplets of OAm for further characterization, except that the TEPA/TEG capped products were dispersed in ethanol.

1. ***Theoretical Methods***

Calculations were performed using dispersion-corrected density functional theory (DFT-D) within the generalized gradient approximation (GGA) of PBE functional as implemented in the DMol^3^ package.^[2-4]^ The stoichiometric slab models of (110), (1$\bar{\text{1}}$0), (001) and (100) are 20.26×20.19×26.73 Å, 19.91×19.80×26.64 Å, 19.04×19.04×26.75 Å, 19.04×25.26×26.76 Å, respectively. We used 1×1×1 k-point grids during the calculation. All the structures were relaxed, in which the convergence tolerance parameters of energy, maximum force, maximum stress and maximum displacement were 2.0 × 10^-5^ eV/atom, 0.004 eV/Å, 0.1 GPa and 0.005 Å, respectively. To separate the periodic image, a sufficiently large vacuum slab of 20 Å was used.

**Supplementary Figures**

**
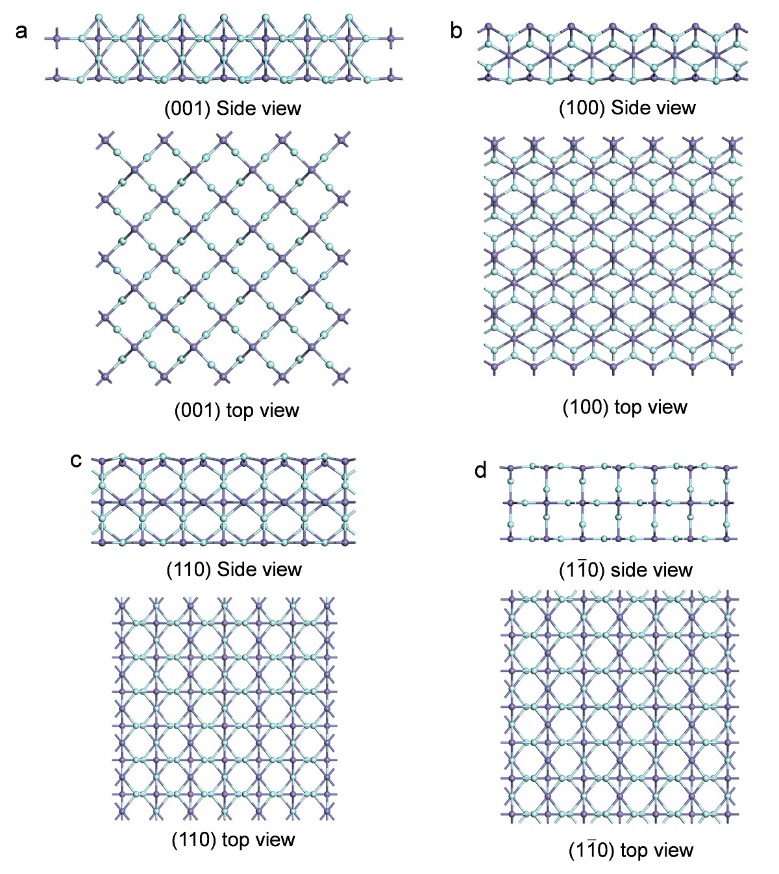
**

**Figure 1.** Slab models used in the DFT calculations. The stoichiometric slab models of (110), (1$\bar{\text{1}}$0), (001) and (100) are 20.26×20.19×26.73 Å, 19.91×19.80×26.64 Å, 19.04×19.04×26.75 Å, 19.04×25.26×26.76 Å, respectively.

**
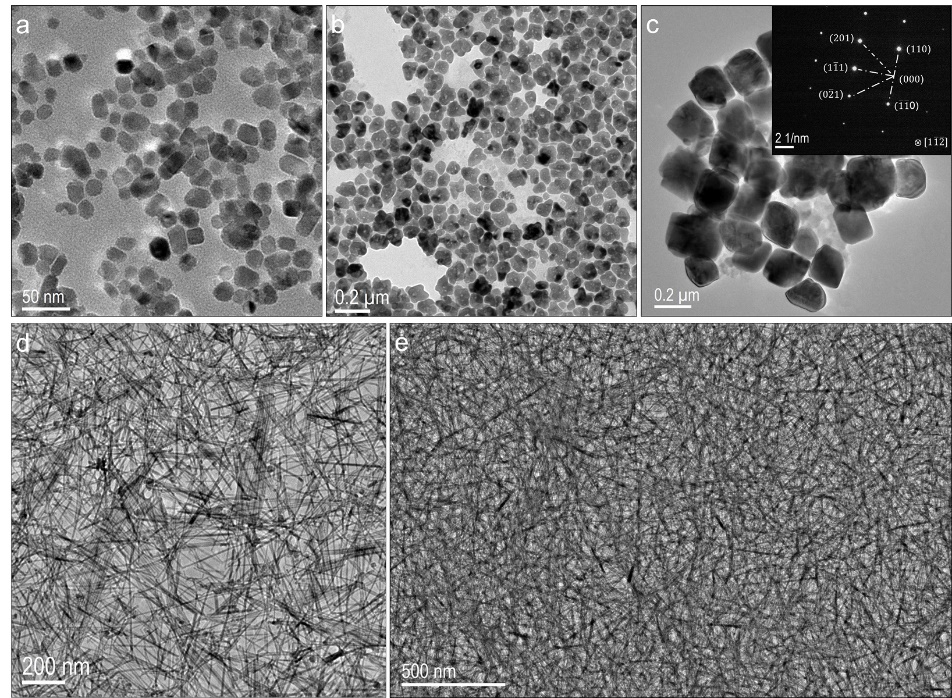
**

**Figure 2.** Bright-field TEM images of varied FeF_2_ nanocrystals. (a-c) 0D FeF_2_ particulates with varied diameters. Inset is the SAED pattern of an isolated particulate. (d-e) 1D FeF_2_ rods and their corresponding assemblies.

**
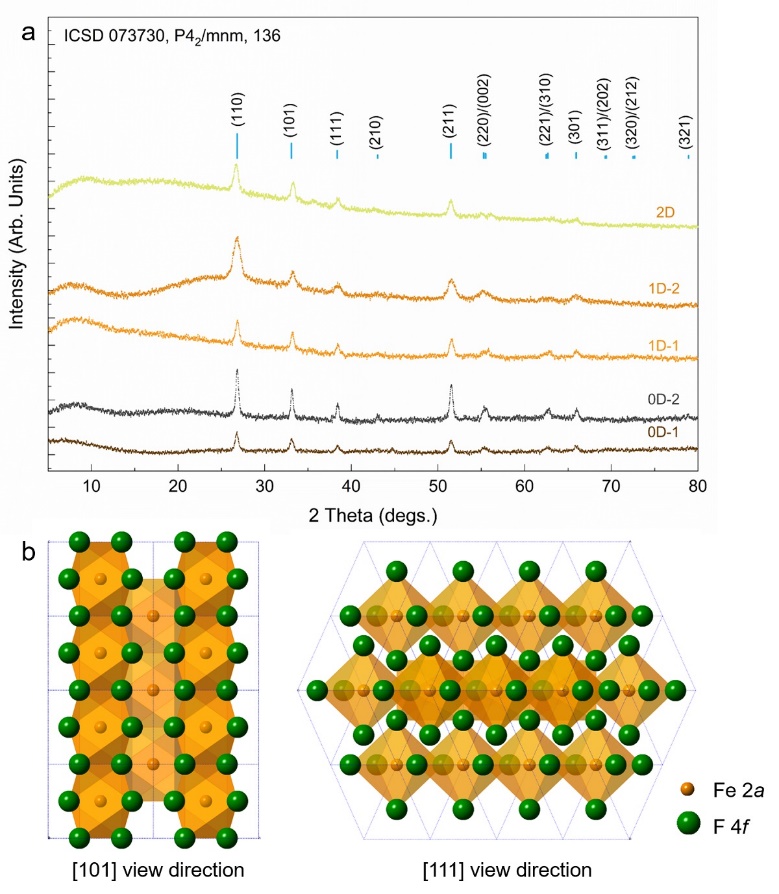
**

**Figure 3.** Structural analysis and crystallographic illustration of FeF_2_ nanocrystals. (a) PXRD analysis of FeF_2_ nanocrystals with varied dimensionalities and diameters. (b) Crystallographic structure viewed from [101] and [111] direction.

**
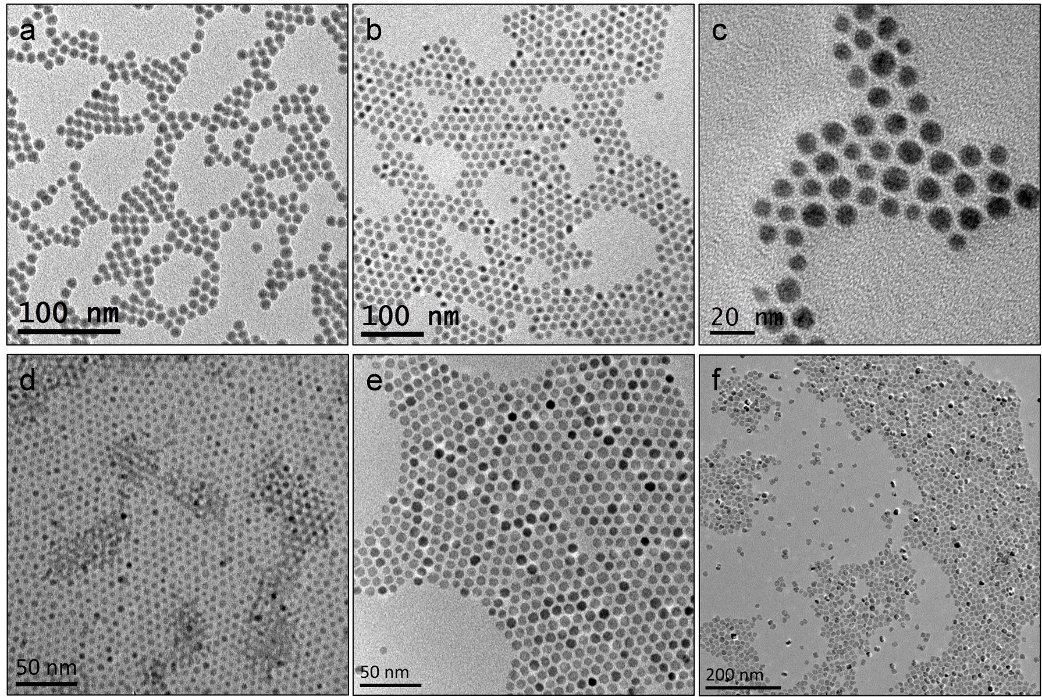
**

**Figure 4.** Bright-field TEM images of metallic and oxides seeds. (a-c) Amorphous and *bcc* structured Fe particulates, and *bcc*-Co particulates (c). (d-f) TEM images of the typical MnO, Fe_3_O_4_ and CoO particulates.

**
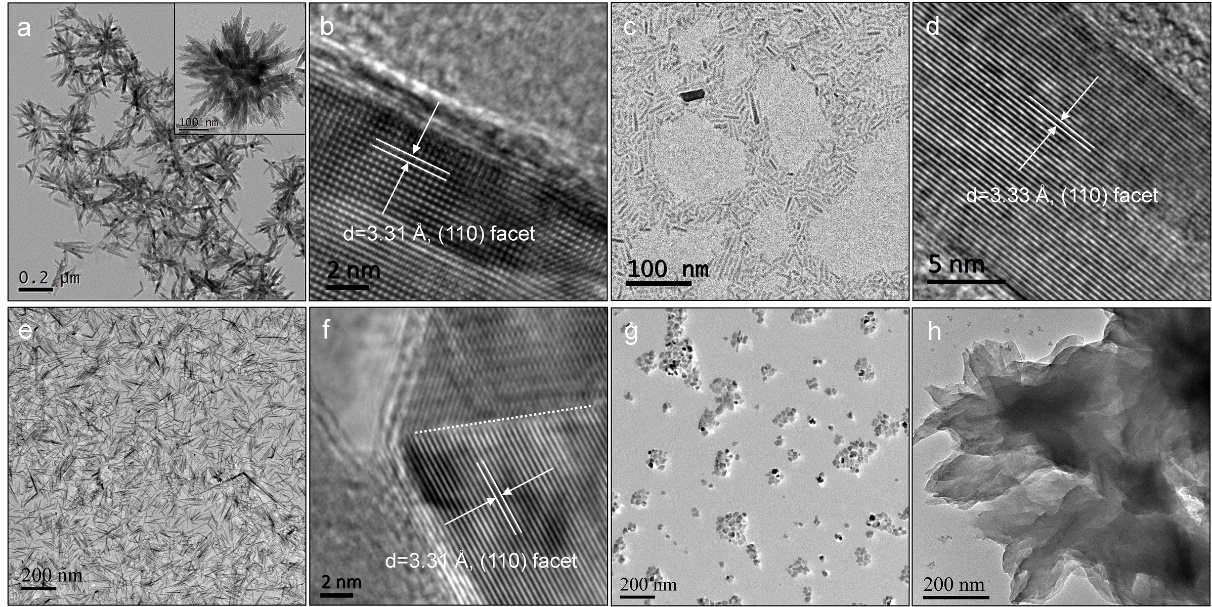
**

**Figure 5.** Bright-field TEM and HRTEM images of CoF_2_ nanocrystals. (a-b) Branched CoF_2_ rods. (c-f) TEM and HRTEM images of the 1D CoF_2_ rods with different aspect ratios. The lattice fringes show evident distortions at the surface area and the existence of twin-crystals. (g-h) 0D particulate and 2D stacked sheets of CoF_2_ nanocrystals.

**
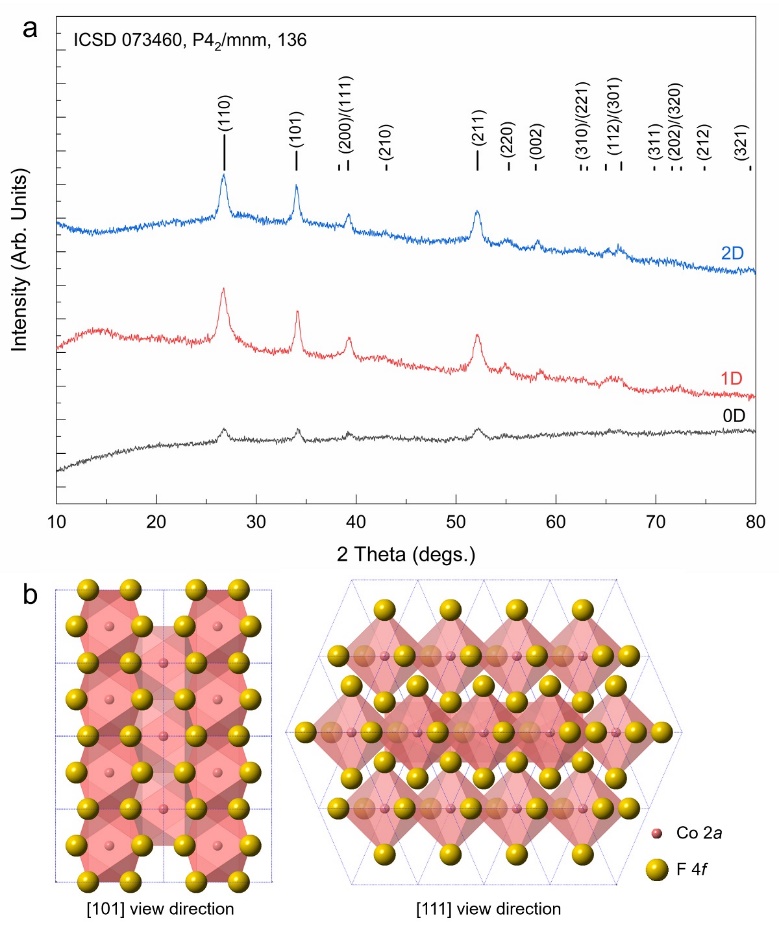
**

**Figure 6.** Structural analysis and crystallographic illustration of CoF_2_ nanocrystals. (a) PXRD analysis of CoF_2_ nanocrystals with varied dimensionalities. (b) Crystallographic structure viewed from [101] and [111] direction.

**
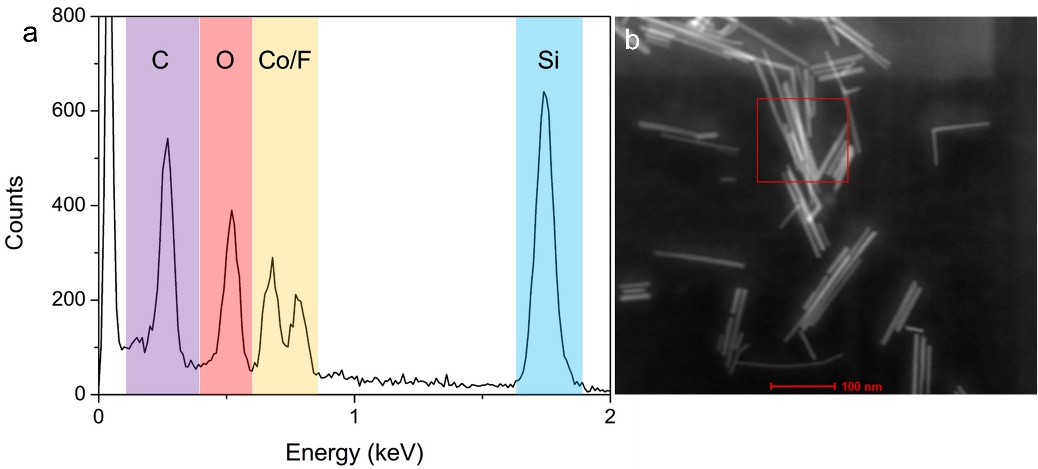
**

**Figure 7.** (a) EDS spectrum of the 1D CoF_2_ rod, which shows the existence of oxygen. (b) STEM image of the selected 1D CoF_2_ rod, the red box shows the area where the EDS spectrum is collected.

**
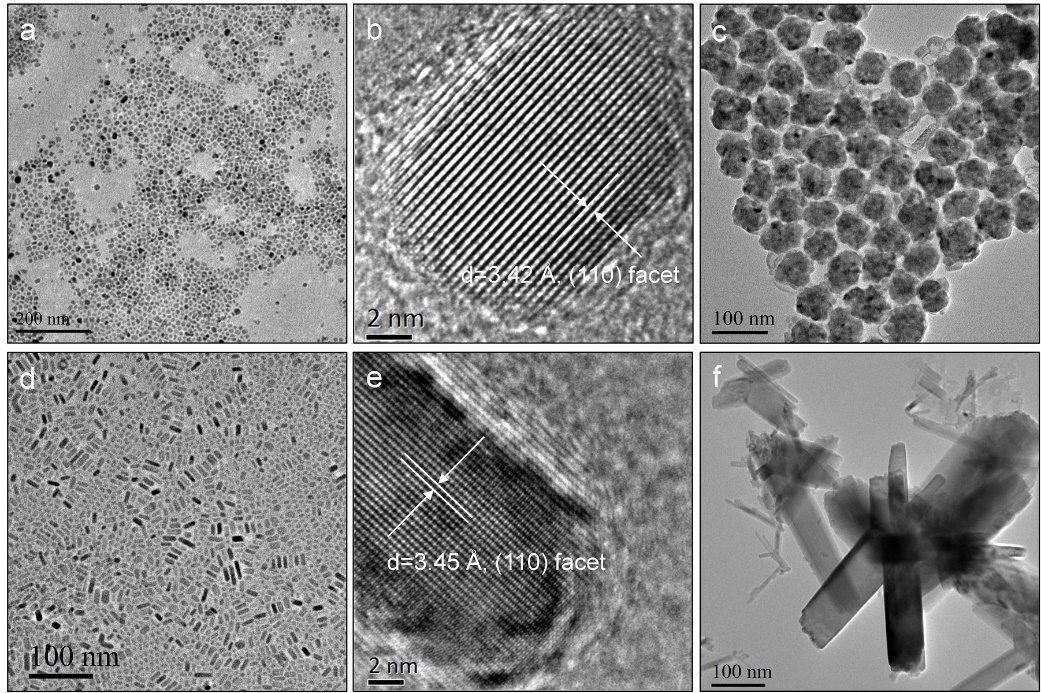
**

**Figure 8.** Bright-field TEM and HRTEM images of MnF_2_ nanocrystals. (a-c) TEM and HRTEM images of 0D MnF_2_ particulates with varied diameters. (d-e) TEM and HRTEM images of the 1D MnF_2_ rods. (f) 2D plate-like MnF_2_ nanocrystals.

**
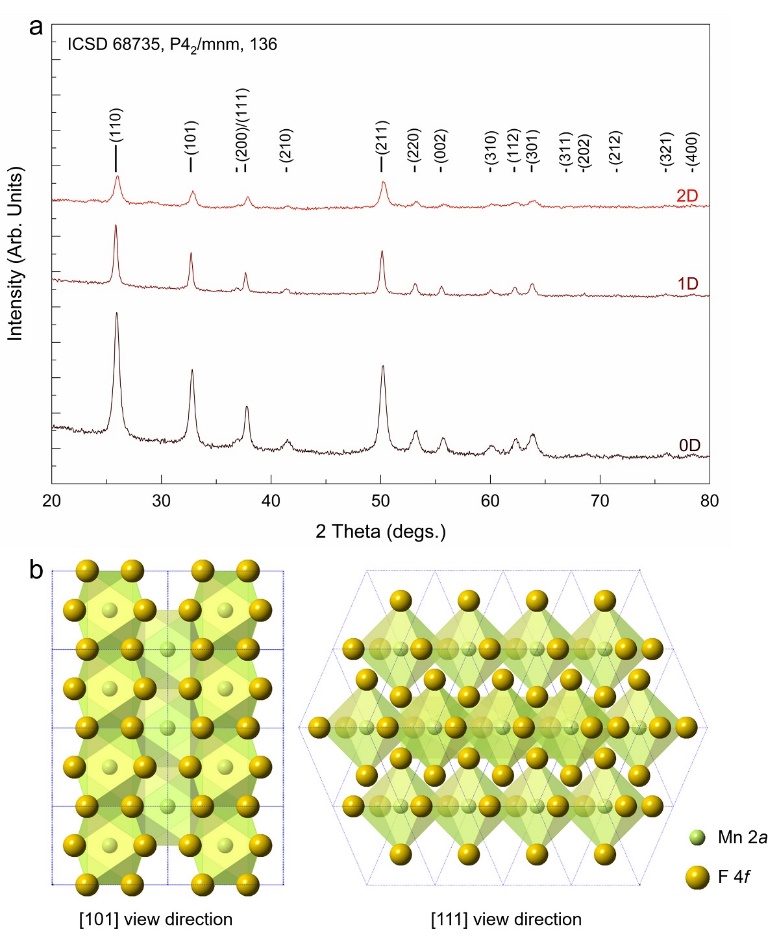
**

**Figure 9.** Structural analysis and crystallographic illustration of MnF_2_. (a) PXRD analysis of MnF_2_ nanocrystals with varied dimensionalities. (b) Crystallographic structure viewed from [101] and [111] direction.

**
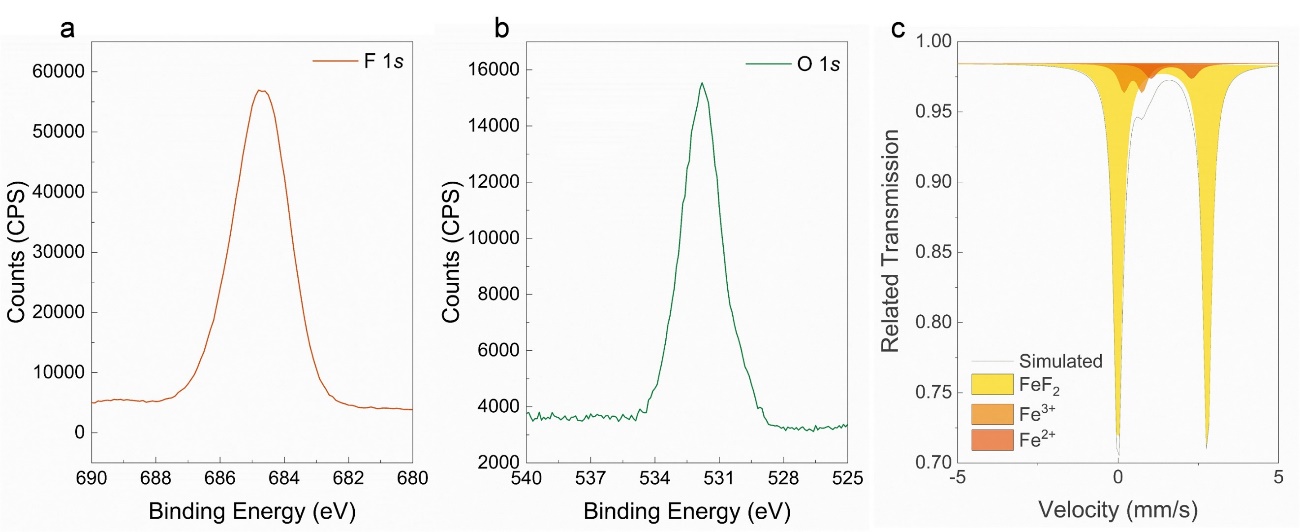
**

**Figure 10.** (a) XPS spectra of F 1*s* and O 1*s*. (b) Mössbauer spectrum of the 42 nm FeF_2_ particulate with three Q.S. doublet fittings.

**Table 1.** Mössbauer hyperfine parameters for FeF_2_ particulates with varying diameters.

| **Sample** | **Portions** | **I.S.^[a]^** | **Q.S.^[b]^** | **L.W.^[c]^** | **Area (%)** | **Note** |
| --- | --- | --- | --- | --- | --- | --- |
| Particulate -220 | S1 | 1.384 (1) | 2.793 (1) | 0.310 (1) | 91.8 | Bulk |
|  | S2 | 0.53 (2) | 0.61 (4) | 0.65 (7) | 8.2 | Fe^III^ |
|  | S3 | - | - | - | 0 | Fe^II^ |
|  |  |  |  |  |  |  |
| Particulate -42 | S1 | 1.384 (1) | 2.783 (1) | 0.322 (2) | 87.7 | Bulk |
|  | S2 | 0.46 (1) | 0.57 (2) | 0.39 (4) | 7.3 | Fe^III^ |
|  | S3 | 1.66 (3) | 1.28 (5) | 0.48 (7) | 5.0 | Fe^II^ |
|  |  |  |  |  |  |  |
| Particulate -16 | S1 | 1.384 (1) | 2.790 (2) | 0.313 (3) | 81.6 | Bulk |
|  | S2 | 0.49 (1) | 0.67 (2) | 0.39 (2) | 13.1 | Fe^III^ |
|  | S3 | 1.6 (2) | 1.0 (2) | 1.0 (4) | 5.3 | Fe^II^ |

[a] I.S.-isomer shift. [b] Q.S.-quadrupole splitting doublets. [c] L.W.-linewidth.

**
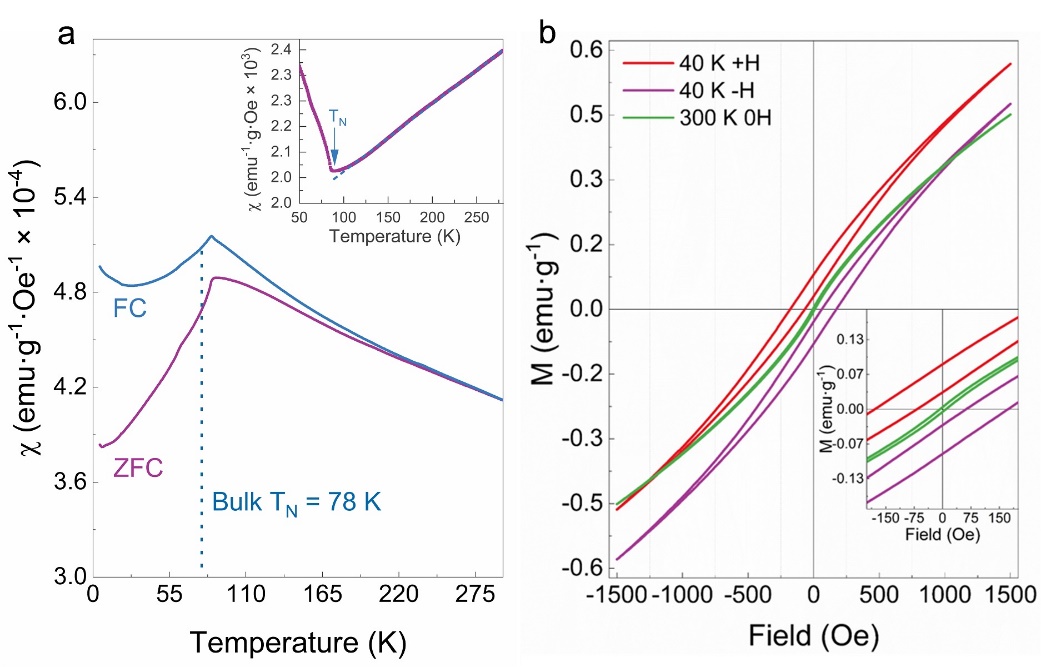
**

**Figure 11.** (a) Magnetization of ZFC (purple) and FC (blue) as function of temperature for 24 ± 1 nm 0D FeF_2_ particulate, the applied field is 50 mT, inset shows that the inverse susceptibility in the high-temperature regime follows Curie-Weiss law *χ* = *C*/(*T*-*Θ_P_*), indicating the presence of robust long-range AFM order in the system. (b) Hysteretic magnetic isotherms of 40 K and 300 K, obtained from dc magnetization measurement with the field up to 1500 Oe under field cooling (± 5 T) processes.

**
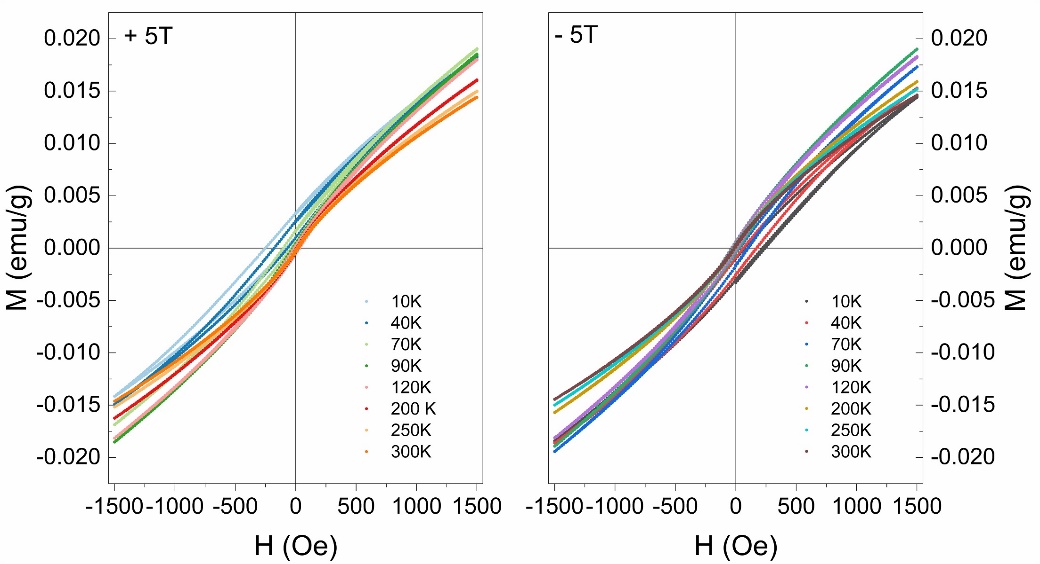
**

**Figure 12.** Magnetic isotherms obtained from dc magnetization measurement with the field up to 1500 Oe under field cooling (± 5 T) processes.

**
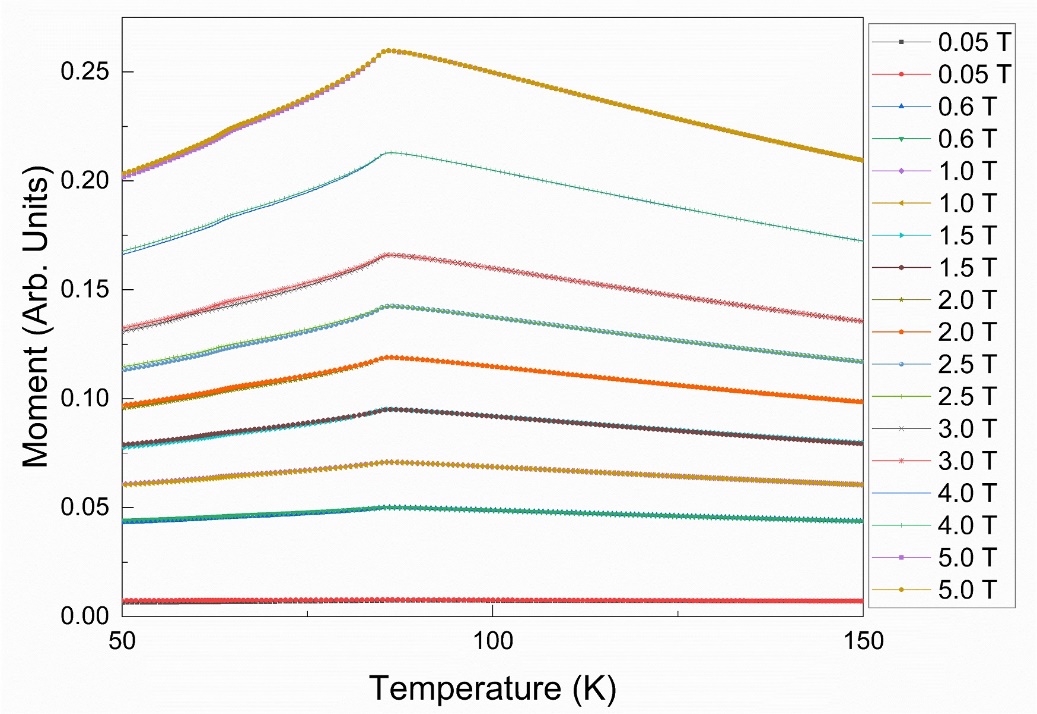
**

**Figure 13.** ZFC and FC magnetization as a function of temperature under different applied fields from 0.05~5.0 T.

**
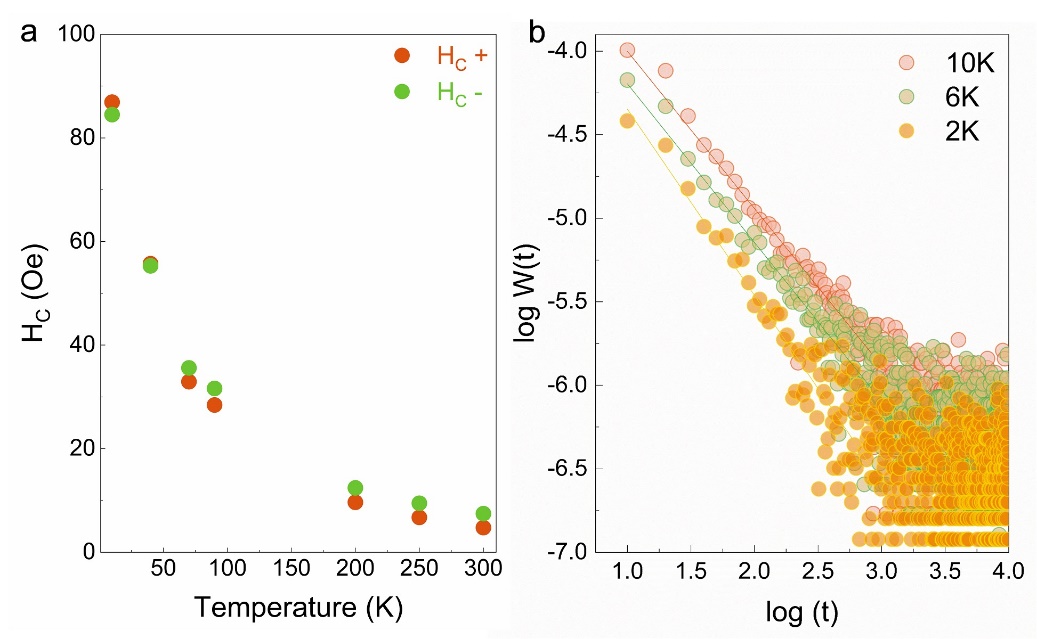
**

**Figure 14.** Static and dynamical analysis of 0D FeF_2_ particulate with a diameter of 24 ± 1 nm. (a) Coercive force *H_C_* as a function of temperature after the field-cooling process (± 5T). (b) The magnetic relaxation rate *W(t)* as a function of time-lapse at 2, 6 and 10 K on a *log_10_-log_10_* presentation. The linear fit is used at the early stage of time-lapse.

**References**

[1]. Z. Yang, T. Zhao, X. Huang, X. Chu, T. Tang, Y. Ju, Q. Wang, Y. Hou, and S. Gao. Modulating the Phases of Iron Carbide Nanoparticles: From a Perspective of Interfering with The Carbon Penetration of Fe@Fe_3_O_4_ by Selectively Adsorbed Halide Ions. *Chem. Sci.,* 2017, 8, 473-481.

[2]. J. P. Perdew, K. Burke, and M. Ernzerhof. Generalized Gradient Approximation Made Simple. *Phys. Rev. Lett.,* 1996, 77 (18), 3865-3868.

[3]. John P. Perdew, J. A. Chevary, S. H. Vosko, Koblar A. Jackson, Mark R. Pederson, D. J. Singh, and Carlos Fiolhais. Atoms, Molecules, Solids, and Surfaces: Applications of the Generalized Gradient Approximation for Exchange and Correlation. *Phys. Rev. B,* 1992, 46 (11), 6671-6687. Erratum: *Phys. Rev. B*, 48, 4978.

[4]. B. Delley. From Molecules to Solids with The Dmol^3^ Approach. *J. Chem. Phys*., 2000, 113, 7756–7764.
